# The effectiveness of community engagement in public health interventions for disadvantaged groups: a meta-analysis

**DOI:** 10.1186/s12889-015-1352-y

**Published:** 2015-02-12

**Authors:** Alison O’Mara-Eves, Ginny Brunton, Sandy Oliver, Josephine Kavanagh, Farah Jamal, James Thomas

**Affiliations:** Social Science Research Unit, UCL Institute of Education, London, UK; Institute for Health and Human Development, University of East, London, UK

**Keywords:** Community engagement, Community participation, Community development, Systematic review, Meta-analysis, Meta-regression, Theoretical model, Public health, Evaluation, Intervention

## Abstract

**Background:**

Inequalities in health are acknowledged in many developed countries, whereby disadvantaged groups systematically suffer from worse health outcomes such as lower life expectancy than non-disadvantaged groups. Engaging members of disadvantaged communities in public health initiatives has been suggested as a way to reduce health inequities. This systematic review was conducted to evaluate the effectiveness of public health interventions that engage the community on a range of health outcomes across diverse health issues.

**Methods:**

We searched the following sources for systematic reviews of public health interventions: Cochrane CDSR and CENTRAL, Campbell Library, DARE, NIHR HTA programme website, HTA database, and DoPHER. Through the identified reviews, we collated a database of primary studies that appeared to be relevant, and screened the full-text documents of those primary studies against our inclusion criteria. In parallel, we searched the NHS EED and TRoPHI databases for additional primary studies. For the purposes of these analyses, study design was limited to randomised and non-randomised controlled trials. Only interventions conducted in OECD countries and published since 1990 were included. We conducted a random effects meta-analysis of health behaviour, health consequences, self-efficacy, and social support outcomes, and a narrative summary of community outcomes. We tested a range of moderator variables, with a particular emphasis on the model of community engagement used as a potential moderator of intervention effectiveness.

**Results:**

Of the 9,467 primary studies scanned, we identified 131 for inclusion in the meta-analysis. The overall effect size for health behaviour outcomes is *d* = .33 (95% CI .26, .40). The interventions were also effective in increasing health consequences (*d =* .16, 95% CI .06, .27); health behaviour self-efficacy (*d =* .41, 95% CI .16, .65) and perceived social support (*d =* .41, 95% CI .23, .65). Although the type of community engagement was not a significant moderator of effect, we identified some trends across studies.

**Conclusions:**

There is solid evidence that community engagement interventions have a positive impact on a range of health outcomes across various conditions. There is insufficient evidence to determine whether one particular model of community engagement is more effective than any other.

**Electronic supplementary material:**

The online version of this article (doi:10.1186/s12889-015-1352-y) contains supplementary material, which is available to authorized users.

## Background

Historically, interventions and actions to promote health were driven by professionals with little or no input from the targeted populations [1]. A more recent movement from practitioners, policymakers, and researchers has been to engage members of the community in public health interventions (e.g., [2,3]). Community engagement has been broadly defined as “involving communities in decision-making and in the planning, design, governance and delivery of services” ([4] p 11). Community engagement activities can take many forms and are usually described in terms of five levels of engagement (from least to most engaged): information-giving, consultation, joint decision-making, acting together, and supporting independent community interests [5].

Community engagement has been advocated as a potentially useful strategy to reduce health inequalities (e.g., [6-8]). Health inequalities are evident where disadvantaged groups (e.g., people with low socioeconomic status, socially excluded people) tend to have poorer health than other members of society [8]. Importantly, health inequalities refer to differences in modifiable health determinants, such as housing, employment, education, income, access to public services, and personal behaviour (e.g., use of tobacco), as opposed to fixed determinants such as age, sex, and genetics. Given that the social determinants of health are potentially modifiable, community engagement interventions to reduce health inequalities have been implemented and evaluated. There are, however, few investigations of whether intervention effects can be directly attributed to the community engagement strategy—most evaluations differ between the intervention and control conditions in more ways than just the engagement of community members.

Previous reviews of the community engagement literature suggest potential social improvements but unclear effects on morbidity, mortality and health inequalities [6,9]. An international literature review for the World Health Organisation found that participatory empowerment has been linked to positive outcomes such as social capital and neighbourhood cohesion for socially excluded groups [6]. However, the author noted that links to health outcomes are more difficult to identify. Similarly, Popay et al.’s rapid review [9] found some evidence for improvements in social capital, social cohesion, and empowerment as a result of community engagement, but little evidence of improvements for mortality, morbidity, health behaviours, or impact on inequalities. The authors found that no studies evaluated the effect of community engagement on outcomes directly, and that evaluations were often carried out too soon in the intervention lifecycle to demonstrate impact.

In summary, it seems that community engagement is likely to have a positive effect on social inequalities [6,9], which might in turn reduce health inequalities [8], although the direct effect on health inequalities is still uncertain [6,9]. This review attempted to examine both direct and indirect pathways to reducing health inequalities through community engagement approaches, by taking a broader approach to the literature than previous reviews and through the use of innovative search processes to identify the dispersed evidence.

## Methods

### Design and definitions

This paper presents the results of a statistical analysis that is one component in a broader project (reported in [10]^a^). The full project was a multi-method systematic review containing four components in addition to the meta-analysis presented here: a map of the evaluative and theoretical literature that describes the scale and range of community engagement interventions; a thematic summary of process evaluations linked to evaluation studies focused on health inequality policy priority areas; an analysis of costs and resources; and a newly developed conceptual framework that brings together the learning from all components of the project. An advisory group that consisted of expert academics and practitioners helped to guide the conceptual focus of the review, including the decision about what studies to include in the meta-analysis.

We use several key terms in this paper. A ‘community’ is a group of people with some common, identified feature, such as geographical location or a socio-demographic characteristic [11,12]. An ‘engagee’ is a member of the community that is involved in the identification, design, and/or delivery of the intervention; engagees are distinct from the intervention ‘participants’, who receive the intervention. The intervention ‘deliverer’ is the person who delivered the intervention, regardless of their status as an engagee or professional [10].

### Aims and research questions

The primary purpose of these analyses is to consider the overall effectiveness of public health interventions that incorporate community engagement strategies, compared with controlled conditions in which no or minimal community engagement is evident (drawing on concepts such as “Arnstein’s ladder” to facilitate judgements here [13]). Effectiveness of the interventions was assessed for health behaviour (e.g., diet, physical activity, smoking habits), health consequence (e.g., change in body mass index, reduction in cholesterol), self-efficacy, perceived social support, and community outcomes (e.g., improvements in the local area). A secondary aim is to explore moderators of the intervention effect, including study characteristics (e.g., country in which the study was conducted), intervention characteristics (e.g., how community engagement was operationalised; and characteristics of the intervention providers), participant characteristics (e.g., age), and features of the evaluations (e.g., risk of bias). These analyses will help us to answer the following questions^b^:

- Do public health interventions that engage members of the community improve health-related outcomes (health behaviours, health consequences, self-efficacy, perceived social support, and community outcomes)?
- Are different approaches to engagement differentially effective?
- Do certain features of the interventions (health topic, universal versus targeted approach, intervention setting, intervention strategy, intervention deliverer, and duration of the intervention) moderate intervention effectiveness?
- Are certain features of the participants (health inequality category, age) related to intervention effectiveness?
- Do features of the evaluation impact observed effectiveness (i.e., is there a risk of bias)?

### Study identification and selection for the meta-analysis

The search syntax strategies used are presented in Appendix A and the detailed screening and inclusion criteria are recorded in the full project report [10] and in a methodology paper [14]. Here we briefly summarise the process which differed from many systematic reviews, because the concepts that we were searching for (i.e. community engagement and inequalities) were not always central concerns of the studies we were looking for – and hence would not appear systematically in their titles, abstracts or keywords. In order to overcome this, we identified systematic reviews of public health interventions, and utilised the structured information in their evidence tables to find relevant studies for our review. Electronic searches thus focused on the identification of systematic reviews (from which we identified primary studies), and electronic searches for primary studies were less extensive than would usually be the case. We estimate that more than a quarter of the studies we included would have been missed using traditional search techniques [14].

We searched the following sources without language restriction for systematic reviews of public health interventions: Cochrane CDSR and CENTRAL, Campbell Library, Database of Abstracts of Reviews of Effects, NIHR Health Technology Assessments programme website, Health Technology Assessments database, and the Database of promoting health effectiveness reviews (DoPHER). Through the identified systematic reviews, we collated a database of primary studies that appeared to be relevant and screened the full-text documents of those primary studies against our inclusion criteria. In parallel, we searched the NHS EED and TRoPHI databases for primary studies which may not have been included in any existing systematic reviews. We also contacted key authors and conducted citation searching of included studies to identify further studies.

Full-text reports of all systematic reviews on public health topics identified through these sources were retrieved; their summary tables were then scanned to locate relevant trials. A secondary screening of titles and abstracts eliminated studies published before 1990 and from non-OECD countries. All full-text reports of relevant trials were subsequently retrieved, screened and included if they:

- Reported primary research;
- Were not a Masters thesis;
- Included intervention outcome and/or process evaluations;
- Focused on community engagement as the main approach;
- Contained a control or comparison group;
- Characterised study populations/reported differential impacts of social determinants of health according to the ‘PROGRESS-Plus’ framework [15]: Place of residence, Race/ethnicity, Occupation, Gender, Religion, Education, Socio-economic position, and Social capital, Plus other variables describing ways in which people may be systematically disadvantaged by discrimination (including sexual orientation, disability, social exclusion, and challenging life transitions such as teenage pregnancy); and
- Reported health or health-related (including cost) effectiveness outcomes and/or process data.

Due to the large number of studies identified for inclusion in the map of community engagement interventions (*n* = 319; see full report for details), and in order to align our work with policy priority areas, we narrowed the scope of health topics included in the meta-analysis by focusing on the policy objective areas identified in the Marmot Review of health inequalities, ‘*Fair Society, Healthy Lives’* [8], which assembled evidence and advised the Department of Health, England on the development of a health inequalities strategy, plus the key modifiable health risks identified in the Marmot Review (smoking, alcohol abuse, substance abuse, and obesity). This led to a final sample of 131 studies.

### Data extraction

Data were extracted on models, approaches, and mechanisms of community engagement; health topic; participant characteristics; geographical and contextual details; costs and resources; and processes (the full data extraction tool is included in the report, [10]). To ensure consistency in interpretation and to minimise error, data extraction was undertaken by researchers working independently in pairs, and then meeting to discuss and resolve any disagreements.

Effect size estimates for participants and engagees (where available) were calculated using standard techniques [16], adjusting for cluster allocation [17] where necessary. Effect size estimates based on continuous data were calculated as the standardised mean difference (represented by *d*), while logged odds ratios were used for binary outcomes. Logged odds ratios were transformed to standardised mean differences using the methods described in Lipsey and Wilson [18] so that the different types of effect size estimates could be included in the same analyses^c^. A positive *d* indicates that participants in the treatment group, on average, scored higher than those in the control group. An effect size estimate of *d* = 1.0 means that participants in the treatment group scored – on average – one standard deviation higher than the control group on the particular outcome measure.

We extracted intervention effectiveness data for the following outcomes:

- Health behaviours. Outcomes extracted were: alcohol abuse, antenatal (prenatal) care, breastfeeding, cardiovascular disease, child illness and ill health, drug abuse, healthy eating, immunisation, injury/safety, parenting, physical activity, smoking cessation, smoking/tobacco prevention, and other captured above
- Health consequences. Outcomes extracted were: cardiovascular disease, child abuse prevention, child illness and ill health, healthy eating, hypertension, injury/safety, mental health, obesity/weight status, and other not captured above
- Participant self-efficacy pertaining to the health behaviours
- Participant social support in relation to the health behaviours
- Community outcomes (e.g. ‘local area improved in the last 3 years’)
- Engagee outcomes (e.g. physical activity levels or health knowledge of the engagee)
- Studies could contribute more than one effect size estimate to the dataset under the following conditions:
- Where there were both immediate post-test and delayed follow-up measures, in order to test the persistence of effects over time; and/or
- Where there were outcomes from more than one of the six outcome types listed above (NB. only one outcome from each of the above categories was extracted); and/or
- Where there were measures of both engagees and public health intervention participants.

As a result, we calculated multiple effect size estimates for some studies: a total of 212 across the 131 studies. Of the 212 effect size estimates, 191 were calculated from post-test measurements and 21 were from follow-up measurements. This paper refers only to the 191 post-test effect size estimates unless otherwise stated; the follow-up measures are only explored in terms of long-term outcomes in the section ‘Maintenance of intervention effects’. Of the post-test effect size estimates, 81 studies (42.4%) only contributed one effect size estimate, and the mean number of effect size estimates per study was 1.77 (*SD* = .79).

### Data analysis

There were sufficient data to undertake statistical analyses for all outcomes except community and engagee outcomes, which are presented in tabular format. The results (effect sizes and standard errors) of individual studies are presented in forest plots by outcome category.

We conducted random effects model analyses (ANOVAs and multiple regressions) with maximum likelihood estimators, following the methods described in [16]. We used SPSS macros written by David Wilson^d^ to run the models. For the homogeneity analyses, between groups *Q*-statistic (*Q*_B_) indicates the extent to which the categories of studies differ from each other; and within groups *Q*-statistic (*Q*_W_) indicates the extent to which the effect size estimates within a category differ from each other. Analyses were conducted separately for post-test measures and follow-up measures. Analyses were also conducted separately for the different outcome categories (health behaviours, health consequences, self-efficacy, and social support). As such, each study only contributed one effect size estimate to each analytical model.

The following variables were included in subgroup analyses (variables are defined in the relevant results sections):

- Theory of change underpinning the intervention
- Single or multiple components to the intervention
- Health topic
- Universal versus targeted approach
- Intervention setting
- Intervention strategy
- Intervention deliverer
- Duration of the intervention
- PROGRESS-Plus group
- Age of participants

Controlled trials were assessed for methodological quality using a modified Cochrane risk of bias assessment tool which is reproduced in Appendix B [17]. An overall risk of bias grading of ‘high’ or ‘low’ was assigned on the basis of assessments of three subscales: selection bias, attrition bias, and selective reporting bias. For a study to be classified as ‘overall low risk of bias’, all three types of bias had to be avoided. In addition to the overall risk of bias, the type of comparison group and the randomisation of participants to conditions were assessed in separate random effects ANOVAs as potential methodological features that might affect the observed effect size estimate; these analyses were conducted separately for each outcome type.

Additional analyses were conducted to explore the following issues:

- Sample size. An un-weighted regression analysis with sample size as a predictor variable was conducted to try to explain heterogeneity in the dataset.
- Direct versus indirect comparisons of community engagement. Most interventions were compared to a comparison condition that differed from the intervention in more ways than just community engagement. For example, they might present a completely alternative intervention, or use a waitlist/delayed treatment control condition. We call these indirect comparisons. Direct comparisons are those in which the only difference between the treatment conditions was the presence or absence of community engagement; e.g., one study [19] compared peer with non-peer led health education using the same healthy eating programme materials. We conducted an ANOVA to compare mean effect sizes of these two types of comparisons.
- Health outcome type. A further concern relates to the breadth of health topics and health outcomes included in the sample of studies, which we combine in the analyses under the umbrella of ‘health behaviour outcomes’. As such, we tested the difference between outcome types (breastfeeding, health service use, healthy eating, physical activity, substance abuse, tobacco use, and other health behaviours).

Finally, we considered the possibility of publication bias. Concerns about publication bias have been raised after observations that research evaluations showing beneficial and/or statistically significant findings are more likely to be published than those that have undesirable outcomes or non-significant findings [17]. If this phenomenon does occur, then reviews of a biased evidence base will draw biased conclusions. Unfortunately, it is difficult to assess publication bias because there is no way of knowing the extent of what has not been published. In this review, the risk of publication bias was assessed visually using a funnel plot with the effect size estimate on the x-axis and the estimate’s standard error on the y-axis.

## Results

### Study selection

**Figure 1:**
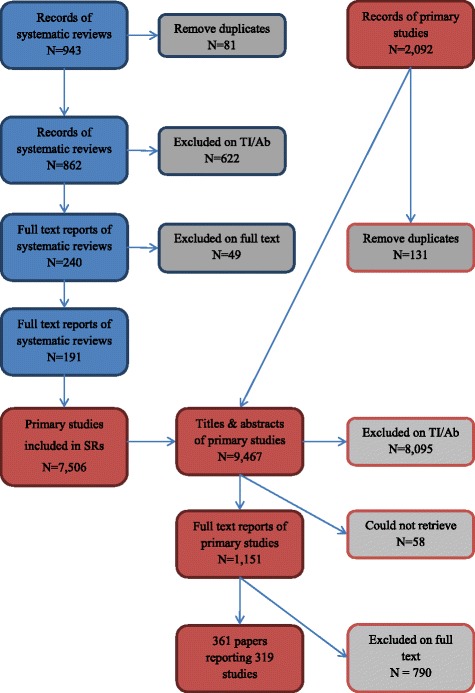
**Flow of systematic reviews (blue) and primary study reports (red) to the map.**

Electronic searches were carried out during July and August, 2011, with supplementary searching continuing during the autumn of 2011. Figure 1 describes the flow of literature through the review process (see Additional file 1 for the full ‘PRISMA checklist’). As outlined earlier, studies were identified for inclusion in the review by searches of databases of systematic reviews and databases of primary research. The flow chart below reflects this two-pronged approach.

We identified 943 records of potentially relevant systematic reviews, 81 of which were duplicate records. Of the 862 unique records, 622 were excluded during assessment of titles and abstracts. Full text copies of 240 systematic reviews were obtained and assessed for eligibility. Seven of these subsequently did not meet minimum methodological standards to be regarded as systematic reviews, and a further forty-two reviews did not include any relevant primary studies. The 7,506 primary studies from the remaining 191 systematic reviews were examined for relevance, an average of 39 studies per review, within a range of three to 547. This process identified 988 eligible studies, all of which were retrieved and re-assessed against our inclusion criteria on the basis of a full-text report.

We also searched TRoPHI and NHS EED databases for reports of primary studies directly, and came across other eligible studies (through recommendations from colleagues or email alerts) before and while working on the review, resulting in 1,961 titles and abstracts to screen after duplicate checking. On the basis of their titles and abstracts, the full texts of 163 of these records were retrieved.

**Figure 2:**
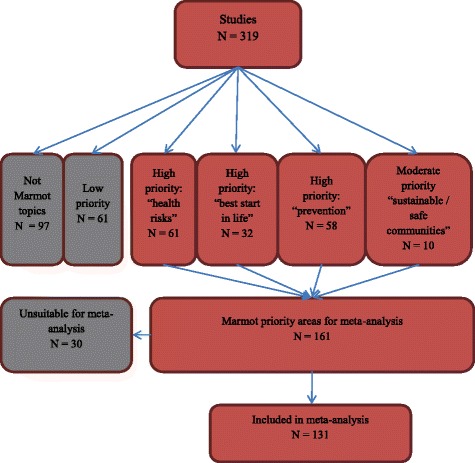
**Prioritisation and selection of studies for the meta-analysis.**

In total, this gave us 1,151 primary study reports to screen on full text, from which a total of 361 reports of 319 studies met our inclusion criteria. After mapping the characteristics of the 319 studies we had identified, we consulted our advisory group and narrowed the focus of the meta-analysis to those studies of high priority areas for the UK, as identified in the Marmot review (8). This is summarised in Figure 2.

### Description of the studies included in the meta-analysis

We present here a summary of the key characteristics of the studies included in this analysis. Full details of all these studies, with a detailed breakdown of the risk of bias assessment, can be found online at reference [10].

### The studies

Of the 131 studies included in the meta-analysis, 113 (86.3%) were conducted in the USA, five (3.8%) were conducted in the UK, five (3.8%) were conducted in Canada, and eight (6.1%) were conducted in other OECD countries. In terms of publication date, 63 (48.1%) were published in the 1990s, 62 (47.3%) in the 2000s, and 6 (4.6%) in 2010 or later.

### The participants

The largest group of studies was classified as being primarily targeted at or delivered to ethnic minority groups (*n* = 56, 42.7%), followed by socioeconomic position (*n* = 34, 26.0%). There was also a large group of studies (*n* = 21, 16.0%) that had multiple PROGRESS-Plus categorisations; the majority of these represented a combination of ethnic minority group status with low income and/or inner-city status. Most of the ethnic minority participants were classified as either ‘Black’, African American, or ‘Hispanic/Latino’.

The studies included a spread of participants across all age groups and most included participants from more than one age group. The majority of the studies (*n* = 79; 60.3%) included young people (age 11–21 years) and/or adults (age 22–54 years; *n* = 65, 49.6%). For sex, 79 (60.3%) studies had mixed sex samples, 49 (37.4%) had predominantly female samples, and three (2.3%) had predominantly male samples.

### The interventions and their evaluation

The interventions were conducted over a range of health topics and settings. The most commonly-targeted health issue was substance abuse (*n =* 18, 13.7%), followed by cardiovascular disease (*n =* 14, 10.7%), breastfeeding (n = 13, 9.9%), obesity prevention / weight reduction (n = 13, 9.9%), smoking cessation (n = 12, 9.2%, public health/health promotion (n = 8, 6.1%) and antenatal care (n = 7, 5.3%). The most common setting for interventions was in the community (*n =* 56, 42.7%). Many interventions were also conducted via media tailored to the participants’ needs (e.g., tailored newsletters or information sheets, *n =* 53, 40.5%) or mass media (*n =* 21; 16%); such interventions could be delivered wherever the participant was located.

Most of the interventions included multiple intervention strategies. The most common strategy was education provision (*n =* 105, 80.2%). Advice (*n =* 71, 54.2%), social support (*n =* 58, 44.3%), and skill development training (*n =* 51, 38.9%) were also common strategies. Interventions were most commonly delivered by peers (*n =* 49, 37.4%) and by community members (*n =* 58, 44.3%).

A variety of comparators were used in the intervention evaluations. The largest group of evaluations employed usual care comparators (*n =* 39, 30%); followed by inactive control (n = 31, 24%), alternative/placebo intervention (n = 28, 21%), waitlist/delayed treatment (n = 16, 12%), matched data from target population (n = 10, 7%), and other/unclear (n = 7, 5%). Thirteen (10%) of the studies examined the effectiveness of community engagement by having a comparison condition that only differed from the intervention by the involvement of community members; for example, an intervention that had the same content but was delivered by a peer in the intervention condition and a health professional in the comparison. Fifty-nine (45%) evaluations used randomisation methods to allocate participants to the intervention or comparison condition. Twenty-six (19.8%) of the evaluations conducted follow-up testing.

### Risk of bias within studies

The risk of bias assessment of the 131 included studies is presented in Additional file 1. On this basis, sixty-nine (52.7%) trials were considered to have an overall low risk of bias and 62 (47.3%) trials were considered to have an overall high risk of bias.

### Results of individual studies

**Figure 3:**
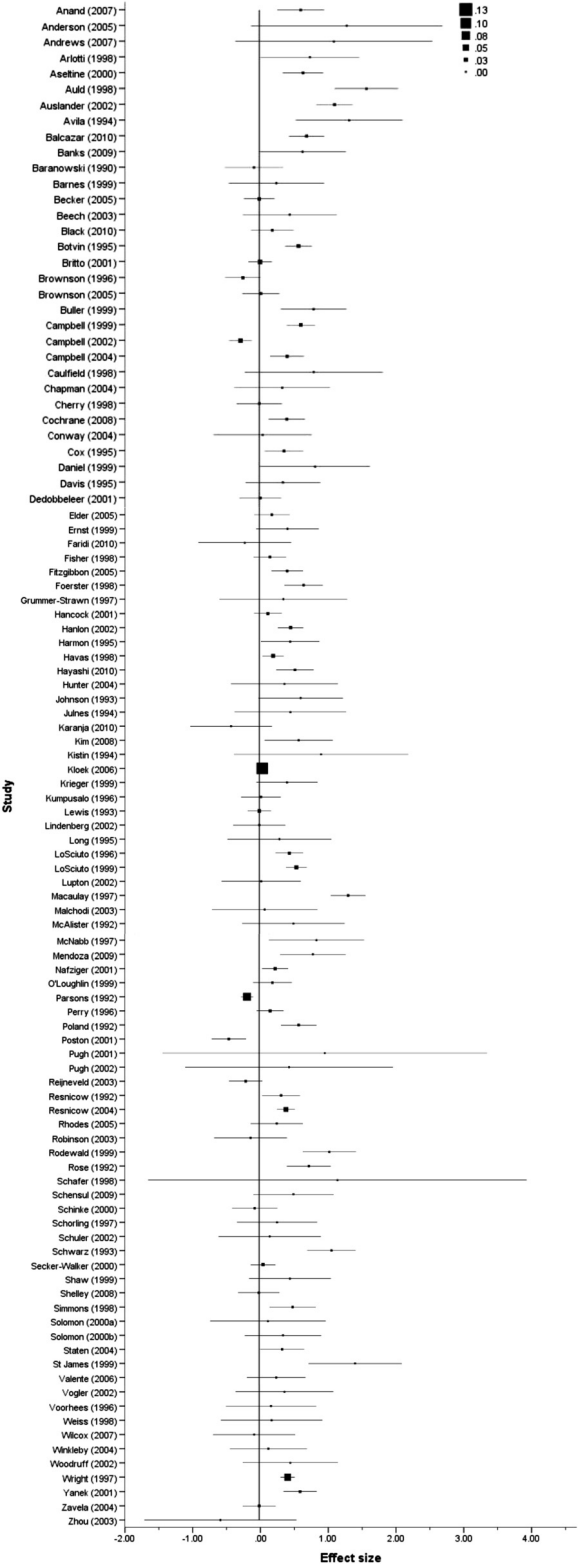
**Forest plot of effect size estimates and standard errors of all studies reporting health behaviour outcomes.**

**Figure 4:**
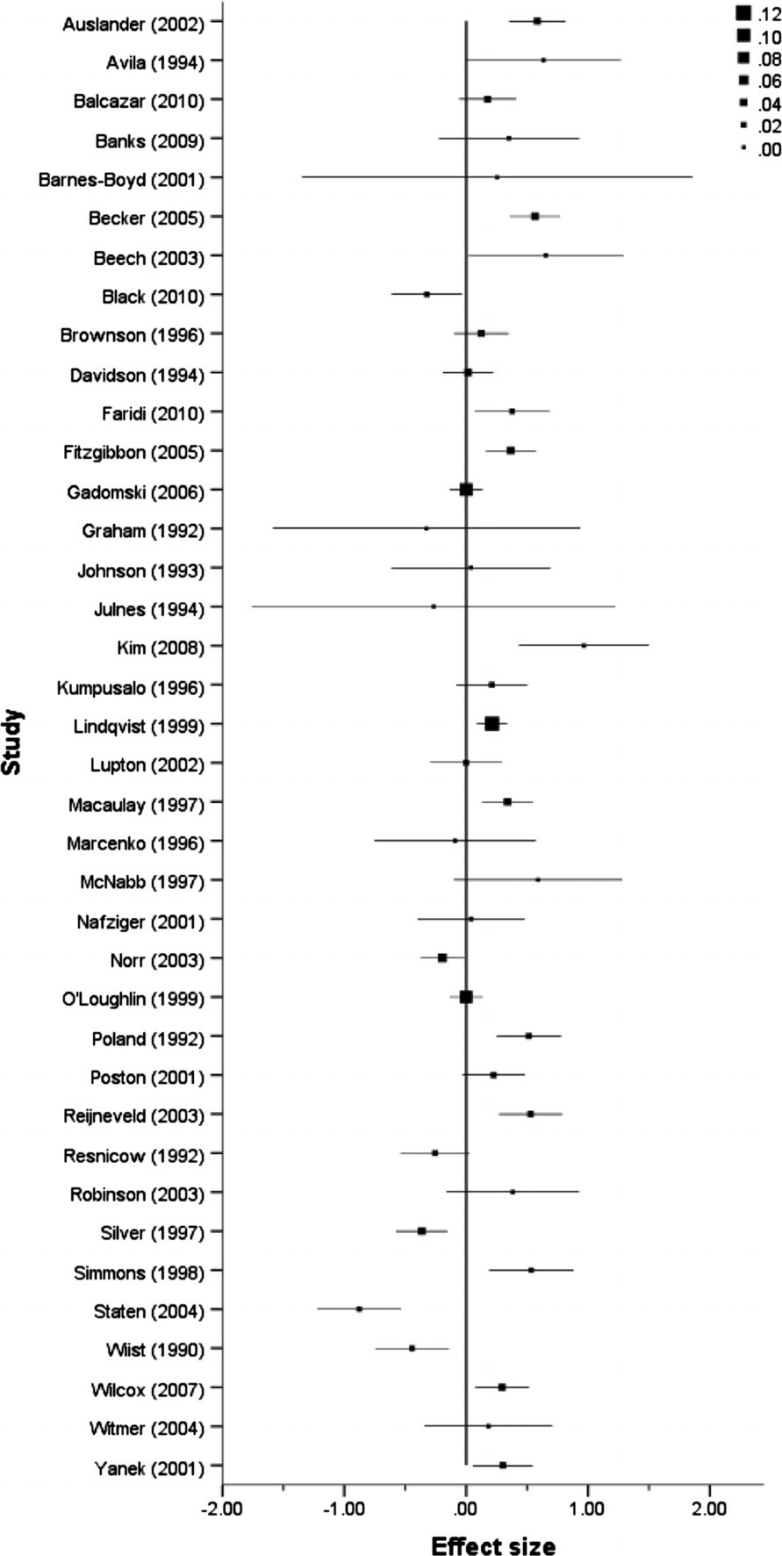
**Forest plot of effect sizes and standard errors of all studies reporting health consequences outcomes.**

**Figure 5:**
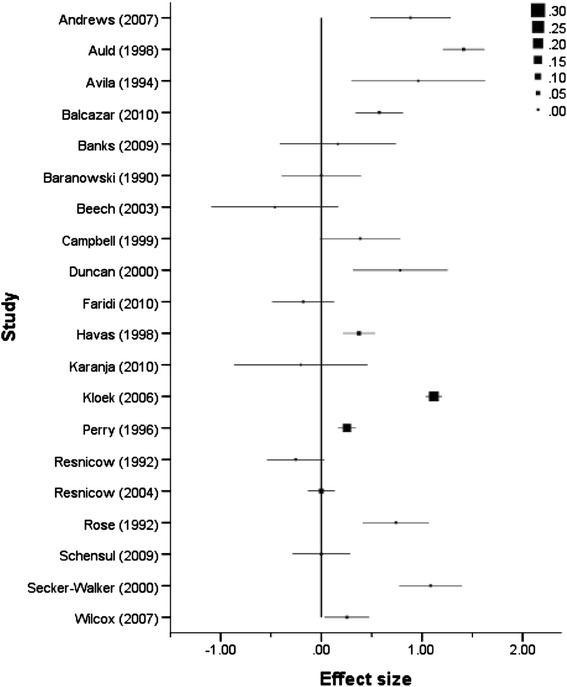
**Forest plot of effect size estimates and standard errors of all studies reporting participant self-efficacy outcomes.**

**Figure 6:**
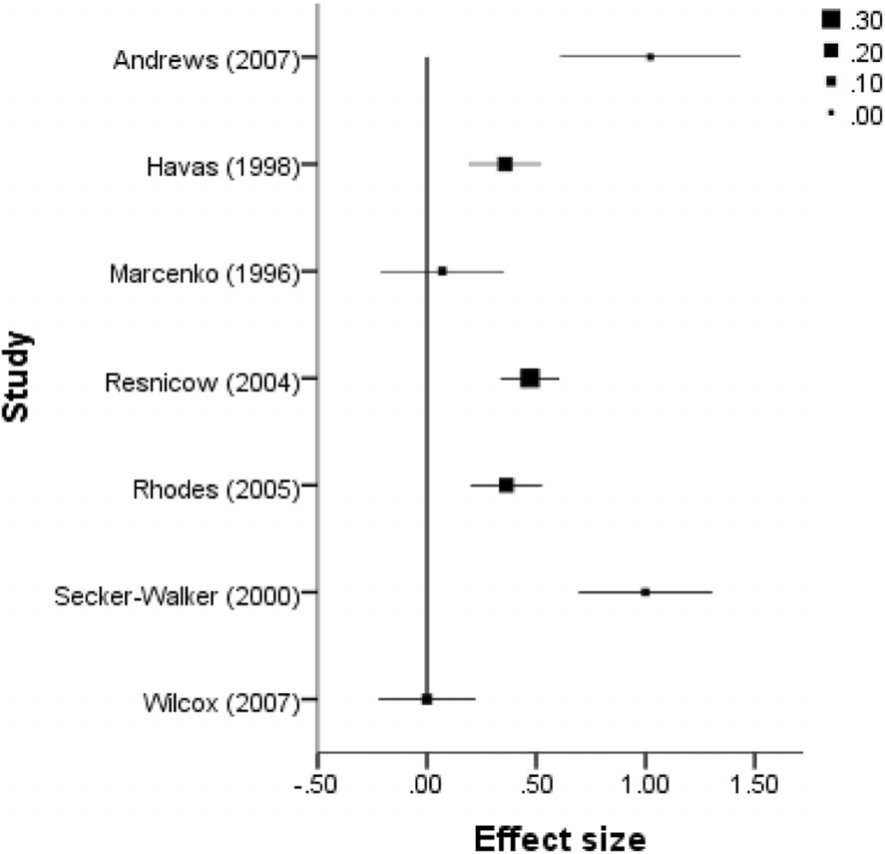
**Forest plot of effect size estimates and standard errors of all studies reporting participant social support outcomes.**

A series of forest plots (Figures 3, 4, 5 and 6) show the effect size estimate, confidence interval, and relative weight for each intervention by outcome type (health behaviours, health consequences, self-efficacy, and social support).

### Results of analyses according to each review question

We now move to the main focus of the results: addressing each of our review questions. We begin with an examination of our overarching question – whether community engagement interventions improve health-related outcomes. We then look to see whether some approaches to community engagement work better than others, whether they work better in some groups than others, and finally examine the relationships between sample size and outcome reported.

### Do public health interventions that engage members of the community improve health-related outcomes?

Interventions were effective across all outcome types (see Tables 1 and 2). There were insufficient effect size estimates for community outcomes and engagee outcomes, so effect size estimates could not be synthesised statistically for these outcomes; we present these effects in Table 1. It is clear from the available outcome data that there are benefits to the community and engagees; all five outcomes are positive and statistically significantly different from a null effect (Table 1).

**Table 1:** **Outcomes description, effect size estimates, and their standard errors for engagee and community outcomes**

**Study**	**Outcome type**	**Outcome description**	**ES estimate**	**Standard error**
***Binary data (logged odds ratios)***				
**Government** **[** 20 **]**	Community outcome	Local area improved in the last three years	1.59***	0.07
**Fried** **[** 21 **]**	Engagee Health	More physical activity at post-test	2.21***	0.37
**Fried** **[** 21 **]**	Engagee Social support/capital/inclusion	Could have used more emotional support from others in the past year	6.57***	0.54
***Continuous data (standardised mean differences)***				
**Ernst** **[** 22 **]**	Community outcome	Connection with health and social services	0.57*	0.24
**Watkins** **[** 23 **]**	Engagee Skills	Lay health workers knowledge	Missing	Missing
**Winkleby** **[** 24 **]**	Engagee Empowerment	Perceptions that advocacy activities would result in changes	1.43***	0.14

*p < .05, ***p < .001. Statistical significance indicates the effect size estimate is significantly different from zero. ES = effect size. ‘Missing’ refers to an outcome that is reported as measured but insufficient data is provided to calculate an effect size.

**Table 2:** **Pooled effect size estimates and heterogeneity for four types of outcomes – random effects model**

				**Heterogeneity**		
**Outcome**	**Pooled effect size estimate**	**95% C.I.**	***n***	***τ*** ^**2**^	***Q*** **statistic**	***I*** ^**2**^
**Health behaviours**	.33***	.26, .40	105	.093	604.62***	82.80
**Health consequences**	.16**	.06, .27	38	.076	196.36***	81.16
**Participant self-efficacy**	.41**	.16, .65	20	.278	480.44***	96.05
**Participant social support**	.44***	.23, .65	7	.067	42.67***	85.94

**p < .01, ***p < .001. Statistical significance indicates the effect size estimate is significantly different from zero. *Note.* 95% CI = 95% confidence interval; *n* = number of effect sizes, *τ* ^2^ = between studies variance.

Table 2 presents the results for the outcomes: health behaviours, health consequences, participant self-efficacy, and participant social support. The pooled effect size estimate across interventions is positive (indicating that the outcomes measured were in favour of the treatment group) and statistically significant (as indicated by the *p*-values and 95% confidence intervals) for these four outcomes. The statistical significance of the pooled effect means that the effect size estimate is significantly different from a null effect in which there are no differences between the intervention group and the comparison group.

There was, however, significant heterogeneity across the four categories of quantitative outcomes observed in Table 2. As such, we conducted moderator analyses to attempt to explain variation in the observed effectiveness of the interventions. These analyses are described in the following sections, but first we examine whether intervention effects lasted beyond the immediate post-test measurement.

#### Maintenance of intervention effects

The maintenance of intervention effects could only be synthesised for health behaviour outcomes because of the lack of follow-up data reported for other outcome types. The pooled effect size estimate at delayed follow-up for the twenty studies that reported health behaviour follow-up outcomes was *d =* .09 (95% CI = −.23, .34), although significant variation (*I*^2^ = 94.43%) suggests that the pooled estimate is not particularly meaningful.

We conducted a meta-regression analysis to attempt to explain the variation. We included post-test effect size estimate as a predictor, so that any variation in the follow-up effect size estimates due to initial intervention effectiveness could be accounted for. We also included the time between the post-test and follow-up measures as a predictor.

The results suggest that post-test effect size estimates do not significantly predict follow-up effect size estimates, although higher post-test effect size estimates tend to be associated with higher follow-up effect size estimates (Table 3). The results also suggest a (just barely non-significant) trend that if the time lapsed after the immediate post-test to follow-up measures is less than a year, the effect size estimate is *smaller* than follow-up measures taken more than a year later. This could suggest some sort of sleeper effect, in which the benefits of the interventions take more than a year to manifest. Another interpretation is that the studies that collected longer term data were those which expected their effects to have greater longevity. The various possible explanations emphasise the need to routinely collect longer term data coupled with process evaluations, to allow testing of these possibilities.

**Table 3:** **Results of the random effects meta-regression analyses examining follow-up effect size estimates**

**Predictor of follow-up effect size estimate**	***B*** **(** ***SE*** **)**
**Constant**	.31 (.19)*
**Post-test effect size estimate**	.37 (.48)
**Less than a year since post-test measure**	-.66 (.34)

*p < .05. Note. B = unstandardised regression coefficient*, SE* = standard error. Q_Model_ (2) = 4.31, *p* = .12, *n* = 17.

### Are different approaches to engagement differentially effective?

#### Theories of change

We ran an analysis to compare the effectiveness of interventions employing one of four different theories of change on health behaviour outcomes, identified in the conceptual synthesis of the broader project (10). The first model proposes that change is facilitated where the health need is identified by the community and they mobilise themselves into action. In the second model, the need for intervention is usually identified by observation from people outside the community, but the views of stakeholders are sought with the belief that the intervention will be more appropriate to the participants’ needs as a result. We identified two main mechanisms through which stakeholder views are sought in the design or planning of the intervention: through collaboration with the community, or through consultation with the community. These two mechanisms are treated as separate models in the analysis. The fourth theory of change model does not necessarily involve the community in the design or planning of the intervention; rather, the focus is on community engagement in the delivery of the intervention (we refer to these throughout this paper as lay-delivered interventions). In this model, change is believed to be facilitated by the credibility, expertise, or empathy that the community member can bring to the delivery of the intervention.

Although there was no overall significant difference between the studies grouped by theory of change, some clear trends emerge (see Table 4). Interventions that engaged the community in the delivery of the intervention had the largest pooled effect size estimate, while interventions that adopted self-mobilisation, design collaboration, or design consultation theories of change (whether implicitly or explicitly) had overall effect size estimates that were similar in magnitude to one another but substantially lower than lay-delivered interventions. Interventions that did not fit into one of the four main theory of change categories (e.g., low engagement in both design and delivery) had the smallest pooled effect size estimate.

**Table 4:** **Results of the random effects ANOVA analyses by theory of change for health behaviour outcomes**

**Theory of change**	**Pooled ES estimate**	**95% CI**	***n***	**Average sample size (** ***SD*** **)**
**Community identified health need**	.31***	.14, .48	17	1067 (226.30)
**Collaboration to design more appropriate intervention**	.32**	.13, .51	16	1924.91 (910.74)
**Consulted to design more appropriate intervention**	.25***	.12, .38	27	848.67 (184.53)
**Lay-delivered to enhance credibility, expertise, or empathy**	.47***	.34, .60	38	309.74 (48.21)
**Other**	.17	-.07, .42	7	757.14 (213.08)

**p < .01, ***p < .001. Statistical significance indicates the effect size estimate is significantly different from zero. *Note.* ES = effect size estimate, 95% CI = 95% confidence interval of the pooled effect size estimate; *n* = the number of effect size estimates in the subgroup; *SD* = standard deviation. Heterogeneity statistics for the meta-analysis: Q_B_ (4) = 7.80, *p* = .10; Q_W_ (100) = 97.63, *p* = .54.

We conducted supplementary analyses to try to explain why the lay-delivered interventions might be more effective. One explanation that we considered was the size of the study. We suspected that the lay-delivered interventions tended to be smaller-scale and usually more likely to be one-on-one, compared to interventions where the community was involved in the design of the intervention. From Table 4, we can see that the mean sample size for studies that only involved the community in the delivery of the intervention is much smaller than in studies based on alternative theories of change. Post hoc analyses of a one-way ANOVA with sample size as the dependent variable and the different theories of change as the factors indicate that the mean sample size for the lay-delivered interventions is statistically significantly smaller than for the interventions in which the community identified the health need.

#### Single and multiple component interventions

In some studies, there were multiple components to an intervention, of which all or only some might have involved community engagement. We categorised the studies into four categories:

- There is only one component to the public health intervention, which involves community engagement in some way
- There are multiple components to the public health intervention, all of which involve community engagement in some way (whether through design, delivery, or evaluation)
- There are multiple components to the public health intervention, only some of which involve community engagement in some way (whether through design, delivery, or evaluation)
- Unclear

There were no significant differences between the four categories for health behaviour outcomes, although there was a trend towards single component interventions having higher effect size estimates (see Table 5).

**Table 5:** **Results of the random effects ANOVA analyses by community engagement in one or more components of the intervention for health behaviour outcomes**

**Components in intervention**	**Pooled ES estimate**	**95% CI**	***n***
**Unclear**	.01	-.33, .35	4
**Only one component**	.42***	.26, .57	30
**All components involve CE**	.34***	.21, .478	31
**Only some components involve CE**	.31***	.20, .43	40

***p < .001. Statistical significance indicates the effect size estimate is significantly different from zero. *Note.* ES = effect size estimate, 95% CI = 95% confidence interval of the pooled effect size estimate; *n* = the number of effect size estimates in the subgroup; CE = community engagement. Heterogeneity statistics for the meta-analysis: Q_B_ (3) = 4.74, *p* = .19; Q_W_ (101) = 96.79, *p* = .60.

### Do certain features of the interventions moderate intervention effectiveness?

We explored a range of characteristics of the interventions, to better understand which types of interventions work best when communities are engaged. The characteristics examined were: health topic, universal versus targeted approach, intervention setting, intervention strategy, intervention deliverer, and duration of the intervention. These variables were selected as they cover key features affecting intervention design, implementation, and resourcing.

#### Health topic

We conducted an analysis to see whether interventions focusing on each of the Marmot Review priority health areas were associated with larger effects. Studies were grouped into three broad categories:

- Modifiable health risks (smoking, alcohol abuse, substance abuse, and obesity)
- Best start in life (antenatal care, breastfeeding, parenting skills, and childhood immunisation)
- Prevention of ill health – topics not captured above (healthy eating, physical activity, general health promotion, injury prevention, cancer prevention, and CVD/hypertension prevention)

We found no significant difference between the three categories for health behaviour outcomes, although there was a trend that impacts were larger for ‘best start in life’ and ‘ill health prevention’ interventions compared to health risks (see Table 6). It is important to emphasise that the pooled effect size estimates for each of the three categories were all significantly different from zero in the positive direction, which indicates that the interventions were generally improving health behaviours, regardless of the health topic.

**Table 6:** **Results of the random effects ANOVA analyses by Marmot themes for health behaviour and health consequences outcomes**

**Outcomes**	**Marmot review theme**	**Pooled ES estimate**	**95% CI**	***n***
**Health behaviours** ^**a**^	Health risks	.24***	.11, .37	34
	Best start in life	.38***	.19, .56	24
	Prevention of ill-health and injury	.38***	.28, .48	47
**Health consequences** ^**b**^	Health risks	.23**	.06, .40	17
	Best start in life	.05	-.29, .39	7
	Prevention of ill-health and injury	.12	-.06, .30	14

**p < .01., ***p < .001. Statistical significance indicates the effect size estimate is significantly different from zero. *Note. n =* the number of effect size estimates in each category, of the predictor variable; ES = effect size; 95% CI = 95% confidence interval. ^a^Q_B_ (2) = 3.01, *p* = .22; Q_W_ (102) = 96.39, *p* = .64. ^b^Q_B_ (2) = 1.23, *p* = .54; Q_W_ (35) = 35.78, *p* = .43.

There were sufficient data to undertake this analysis for health consequence outcomes as well. As with health behaviours, the difference between the three categories was non-significant, although there was a trend in which interventions targeting the best start in life had a smaller pooled effect size estimate than those targeting ill health prevention, which in turn had a smaller pooled effect size estimate than those targeting the modifiable health risks. In contrast to health behaviour outcomes, only the health risks category had a pooled effect size estimate that was significantly different from zero for health consequences outcomes. In other words, there was no evidence that interventions targeting best start in life or the prevention of ill-health had a significant impact on health consequence outcomes.

#### Universal versus targeted approach

In this review, we defined universal interventions as those delivered to large groups, such as a city- or area-wide initiative, and as such may have been exposed to participants that could not be categorised according to the PROGRESS-Plus framework. In contrast, a targeted intervention was deliberately delivered to participants that met specific criteria, such as belonging to a certain ethnic group. There were no significant differences between universal and targeted interventions for health behaviour outcomes (see Table 7). There was a trend towards larger effect size estimates for universal interventions compared to targeted interventions.

**Table 7:** **Results of the random effects ANOVA analyses comparing universal and targeted interventions for health behaviour outcomes**

**Universal or targeted**	**Pooled effect size estimate**	**95% CI**	***n***
**Universal**	.43***	.19, .67	9
**Targeted**	.32***	.24, .40	96

***p < .001. *Note. n =* the number of effect size estimates in each category of the predictor variable; 95% CI = 95% confidence interval. Q_B_ (1) = .70, *p* = .40; Q_W_ (103) = 97.60, *p* = .63.

#### Intervention setting

We found that interventions delivered (whole or in part) in community settings had a significantly smaller pooled effect size estimate for health behaviour outcomes than interventions not conducted in community settings (e.g., in the home, in healthcare settings) (see Table 8).

**Table 8:** **Results of the random effects ANOVA analyses comparing interventions conducted in community settings with non-community settings for health behaviour outcomes**

**Intervention setting**	**Pooled effect size estimate**	**95% CI**	***n***
**Community setting**	.25***	.15, .35	57
**Not community setting**	.42***	.31, .52	48

***p < .001. Statistical significance indicates the effect size estimate is significantly different from zero. *Note.* 95% CI = 95% confidence interval; *n =* the number of effect size estimates in each category, of the predictor variable. Q_B_ (1) = 5.29, *p* < .05; Q_W_ (103) = 96.54, *p* = .66.

#### Intervention strategy

We focused on five particular intervention strategies that were chosen for their prevalence or substantive interest: education, skill development or training, social support, incentives, or access to health resources or services. The results did not indicate any of these intervention strategies were significantly associated with health behaviour outcomes (Table 9). The results indicate that interventions employing incentives or skill development strategies tend to have higher effect size estimates than other strategies, while interventions with education approaches tend to be the least effective.

**Table 9:** **Results of the random effects meta-regression analyses comparing intervention strategies for health behaviour outcomes**

**Intervention strategy**	***B*** **(** ***SE*** **)**	**Mean effect size estimate**
**Constant**	.37 (.10)*	.37
**Education**	-.15 (.10)	.22
**Skill development or training**	.12 (.08)	.49
**Social support**	.05 (.08)	.42
**Incentives**	.10 (.12)	.47
**Access to resources or services**	.01 (.08)	.38

*p < .05. Note. Interventions can have more than one intervention strategy type; the categories are not mutually exclusive. *B* = unstandardised regression coefficient*, SE* = standard error. Q_Model_ (5) = 5.80, *p* = .33. *R* ^2^ = .06, *N* = 105.

#### Intervention deliverer

We focused on four types of intervention provider: community members, peers, health professionals, and educational professionals. These were the people who most commonly provided the intervention and reflect a range of lay people and professionals. These four types of intervention provider did not explain a significant amount of the variation in the effect size estimates of health behaviour outcomes (see Table 10). However, interventions with health professionals involved in the delivery of the intervention tended to have smaller effect size estimates than other types of provider, while those involving educational professionals tended to have larger effect size estimates than other types of provider. It is worth noting that this does not mean that interventions delivered by health professionals caused harm to the participants, as the effects were still positive overall.

**Table 10:** **Results of the random effects meta-regression analyses comparing different intervention deliverer types for health behaviour outcomes**

**Intervention deliverer**	***B*** **(** ***SE*** **)**	**Mean effect size estimate**
**Constant**	.34 (.08)*	.34
**Community member**	-.03(.09)	.31
**Peer**	.03 (.09)	.37
**Health professional**	-.10 (.09)	.24
**Educational professional**	.08 (.10)	.42

*p < .05. Note. Interventions can have more than one intervention deliverer type; the categories are not mutually exclusive. *B* = unstandardised regression coefficient*, SE* = standard error. Q_Model_ (4) = 2.26, *p* = .69. *R* ^2^ = .02, *N* = 105.

By running a reduced model in which we only explored the relative effectiveness of interventions involving community members, peers, or other intervention providers, we were able to test the effectiveness of the interventions by deliverer type for health consequences and participant self-efficacy outcome (see Table 11). For health consequences, we can see a trend towards interventions with community members being more effective than those without community members; however, the results of this model suggest that this is not a significant predictor of effect size estimate.

**Table 11:** **Results of the random effects meta-regression with peer and community intervention deliverers as predictors of intervention effectiveness for health consequences outcomes and self-efficacy**

**Outcome**	***B(SE)*** **constant**	***B(SE)*** **Community member**	***B(SE)*** **Peer**	***n***	***R*** ^**2**^	**Model homog.** ***Q*** **(** ***p*** **-value)**
Health consequences	.06 (.11)	.17 (.13)	.08 (.14)	38	.04	1.70 (*p* = .43)
Participant self-efficacy	.51 (.21)*	-.17 (.23)	.00 (.24)	20	.03	.58 (*p* = .75)

*p < .05. Note. Interventions can have more than one intervention deliverer type; the categories are not mutually exclusive. *B* = unstandardised regression coefficient; *SE* = standard error of the regression coefficient; *n* = the number of effect size estimates included in the analysis; *R* ^2^ = the amount of variance explained by the model, where an *R* ^2^ of .04 represents 4% of the variance in the effect size estimates explained by the model; and Model homog. = homogeneity Q-test value for the model, where a significant value indicates that the model explains significant variability in the effect size estimates.

The reverse trend is apparent for self-efficacy outcomes: interventions delivered by community members are associated with smaller effect size estimates. Again, intervention deliverer was not a significant predictor of self-efficacy effect size estimates.

#### Duration of the intervention

We tested whether the duration of the intervention was associated with the effect size estimates for health behaviours, health consequences, and self-efficacy outcomes. Because duration was not normally distributed, we used two approaches to testing this variable. For health behaviour outcomes, the data were normalised using a logarithmic transformation. For health consequences and self-efficacy outcomes, the data were still non-normal after log transformation, and so we created a categorical variable of short, medium, and long duration interventions.

For health behaviour outcomes, the duration of the interventions is a statistically significant predictor of the effect size estimate: the longer the intervention, the smaller the effect size estimate (see Table 12). For health consequences outcomes, the categories were not significantly different from each other in terms of the pooled effect size estimate, although shorter interventions tended to have larger effects; this is the opposite trend than observed for health behaviours (see Table 13). For self-efficacy outcomes, there were no trends and the variable was not a significant moderator of effect size estimate (see Table 13).

**Table 12:** **Results of the random effects meta-regression with duration of the intervention as a predictor of health behaviour outcomes**

**Intervention duration**	***B(SE)***
**Constant**	.59 (.14)
**Duration**	-.07 (.04)*

*p < .05. Note. B = unstandardised regression coefficient; *SE* = standard error of the regression coefficient. Duration in weeks was normalised using the log transformation before analysis. Q_Model_ (1) = 3.76, *p* < .05. *R* ^2^ = .04, *N* = 100.

**Table 13:** **Results of the random effects ANOVA analyses comparing intervention duration categories for health consequences and self-efficacy outcomes**

**Outcome**	**Intervention duration**	**Pooled ES estimate**	**95% CI**	***n***
**Health consequences** ^**a**^	Less than 6 months	.36**	.16, .57	13
	6 Months to 23 months	.09	-.07, .26	16
	2 or more years	.06	-.16, .28	8
**Participant self-efficacy** ^**b**^	Less than 6 months	.41*	.01, .81	7
	6 Months to 23 months	.41*	.00, .82	6
	2 or more years	.48*	.08, .88	6

*p < .05, **p < .01. *Note.* ES = effect size; 95% CI = 95% confidence interval; *n =* the number of effect size estimates in each category, of the predictor variable. ^a^Q_B_ (2) = 5.20, *p* = .07. Q_W_ (34) = 35.19, *p* = .41. ^b^Q_B_ (2) = .07, *p* = .96. Q_W_ (16) = 18.94, *p* = .27.

### Are certain features of the participants (PROGRESS-Plus group, age) related to intervention effectiveness?

In our review, groups that could be classified as potentially disadvantaged in terms of health included: socio-economic status/position, ethnic minority status, 'at-risk' or 'high risk' young people experiencing social exclusion or life transitions (including pregnant and parenting adolescents), and place of residence (inner-city or rural) in which there was evidence of being medically underserved. There were also many studies with indistinguishable multiple health inequalities (e.g., both low income and ethnic minority status). There were no significant trends by group, although interventions targeted at people that were disadvantaged due to place of residence was the only group that had a pooled effect size estimate that was not significantly different from zero (see Table 14). In other words, there is no clear effect of interventions targeted at people on the basis of their place of residence, although this is likely due to the heterogeneity in the six studies in this category. All other groups had pooled effect size estimates that were significantly different from zero, and interventions targeted at people of low socioeconomic position tended to be particularly effective.

**Table 14:** **Results of the random effects ANOVA analyses by PROGRESS-Plus group for health behaviour outcomes**

**Progress-plus group**	**Pooled ES estimate**	**95% CI**	***n***
**Socio-economic status/position**	.41***	.26, .56	29
**Ethnicity**	.33***	.23, .44	44
**'At-risk' or 'high risk' young people, including pregnant/parenting teenagers**	.45**	.17, .73	6
**Place of residence**	.11	-.16, .38	6
**Multiple health inequalities**	.28**	.12, .45	20

**p < .01, ***p < .001*. Note. n =* the number of effect size estimates in each category of the predictor variable; ES = effect size; 95% CI = 95% confidence interval. Q_B_ (4) = 4.72, *p* = .32; Q_W_ (100) = 96.65, *p* = .58*.*

A separate analysis revealed that age groups targeted in the intervention were not significantly associated with intervention effectiveness for health behaviour outcomes (see Table 15). However, there was a trend such that health behaviour effect size estimates tended to be smaller when the intervention targeted the general population.

**Table 15:** **Results of the random effects ANOVA analyses comparing interventions targeted at different age groups for health behaviour outcomes**

**Age groups targeted**	**Pooled ES estimate**	**95% CI**	***n***
**General population**	.22***	.11, .34	38
**Children or young people (0–17)**	.37***	.25, .50	32
**Young people and adults (11–54)**	.36***	.17, .56	19
**Adults (18+)**	.47***	.29, .64	16

***p < .001*. Note.* ES = effect size; 95% CI = 95% confidence interval; *n =* the number of effect size estimates in each category, of the predictor variable. Q_B_ (3) = 5.97, *p* = .11; Q_W_ (101) = 97.16, *p* = .59.

#### Do features of the evaluation (risk of bias) impact observed effectiveness?

We explored the potential risk of bias by considering three methodological features of studies: the type of comparison group, randomisation of participants to conditions, and the overall risk of bias of the study. As described in the methods section, overall risk of bias is a combined measure of the degree of risk of bias on the three subscales: selection bias, attrition bias, and selective reporting bias.

For all four outcome types (health behaviours, health consequences, self-efficacy, and social support), the analyses revealed no significant moderators of the effect size estimates. The results are presented in Table 16. As such, we can be reasonably confident that there is no systematic bias in the effect size estimates due to methodological characteristics of the evaluation design.

**Table 16:** **Homogeneity results for different potential risk of bias variables on four outcome types**

**Outcome**	**Risk of bias variable**		**Model homogeneity**	
		***k***	**Between groups Q**	**Within groups Q**
			**(** ***p*** **-value)**	**(** ***p*** **-value)**
Health behaviours	Comparison group type	7	7.71 (*p* = .26)	97.14 (*p* = .51)
	Random allocation	3	.14 (*p* = .93)	95.60 (*p* = .63)
	Overall low risk of bias	2	1.27 (*p* = .26)	97.45 (*p* = .64)
Health consequences	Comparison group type	-	Insufficient data	
	Random allocation	-	Insufficient data	
	Overall low risk of bias	2	.18 (*p* = .67)	36.66 (*p* = .44)
Participant self-efficacy	Comparison group type	-	Insufficient data	
	Random allocation	-	Insufficient data	
	Overall low risk of bias	2	1.68 (*p* = .19)	20.33 (*p* = .31)
Social support	Comparison group type	-	Insufficient data	
	Random allocation	-	Insufficient data	
	Overall low risk of bias	2	.04 (*p* = .85)	7.19 (*p* = .21)

Note. *k =* number of categories in the moderator variable; Between groups Q indicates the extent to which the categories of studies differ from each other; and within groups Q indicates the extent to which the effect size estimates within a category differ from each other.

### Additional analyses

#### Sample size

**Figure 7:**
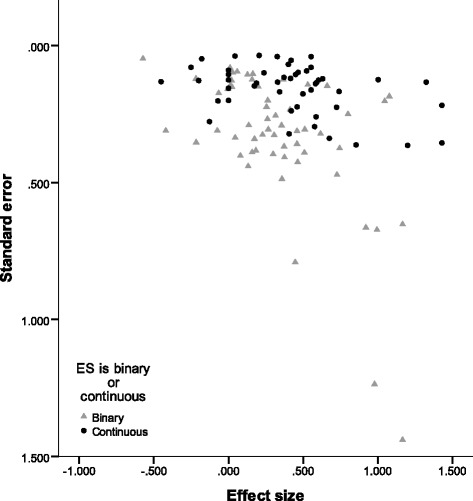
**Plot of effect size estimates by their standard errors, with different markers for effect size estimates based on binary and continuous data.**

One phenomenon that appeared to be related to the effect size estimates was the size of the study, as indicated by the funnel plot in Figure 7. To explore whether the sample size might explain some of the variation in the effect size estimates, we conducted a post hoc un-weighted meta-regression^e^. This model tested whether the log of the sample size of each study predicted the size of the effect for health behaviour outcomes. The results indicated that, although sample size was not a significant predictor of the effect size estimate (*B* = −.10, *SE* = .08), it explained about 10% of the variance in the effect size estimates (as indicated by the model *R*^2^). As such, it is likely that sample size accounts for some of the heterogeneity observed amongst the effect size estimates. Our discussion of the relationship between the theory of change and sample size above (see also Table 4) might suggest that sample size is confounded with other explanatory variables.

### Sensitivity analyses

We tested whether there was a difference between studies that directly tested community engagement compared with indirect comparisons. Two important features are relevant to determining whether it makes sense to combine these outcomes: the between group heterogeneity statistic and the direction of each subgroup’s pooled effect size estimate. The results of the analysis were not statistically significant (which was unsurprising given the small number of studies with direct comparison evaluation approaches; Q_B_ (1) = .01, *p* = .93). The group means suggest no trends in either direction: the pooled effect size estimate was .34 for studies with a direct comparison and .33 for indirect comparisons. This analysis suggests that including both direct and indirect comparisons in the analyses is not likely to be a source of bias.

We also tested the difference between outcome types (breastfeeding, health service use, healthy eating, physical activity, substance abuse, tobacco use, and other health behaviours). The between-group heterogeneity statistic indicates that the groups are not statistically significantly different from each other (Q_B_ (6) = 12.27, *p* = .06). The pooled effect size estimate for each group is statistically significantly different from zero in the positive direction. Although there is some variation in the magnitude of effects, these results do not suggest that we should be concerned about combining these outcomes in the analyses on the basis of statistical differences.

### Risk of publication bias

In Figure 7, the effect size estimates are plotted against their standard errors for both continuous and binary outcomes. From the figure, we can see that larger effect size estimates (in terms of magnitude) typically have larger standard errors; that is, larger effects are associated with more variability. This can indicate a potential for publication bias.

We believe, however, that our sampling frame may help protect us from publication bias. By identifying studies primarily through systematic reviews that have taken measures to protect against publication bias (e.g., searching grey literature), our set of studies includes many reports that would not be subject to the presumed publication bias associated with journal articles.

## Discussion

Overall, public health interventions using community engagement strategies for disadvantaged groups are effective in terms of health behaviours, health consequences, health behaviour self-efficacy, and perceived social support. These findings appear to be robust and not due to systematic methodological biases. The small group of studies that measured longer term outcomes were heterogeneous, although effects generally are smaller than at post-test. There are also indications from a small number of studies that community engagement interventions can improve outcomes for the community and engagees.

We caveat these overall statements with the observation that there is significant variation in the intervention effectiveness; some interventions were more effective than others, and not all interventions benefited the participants. We tested a set of pre-determined variables that we hoped might explain this variance and address the research questions posed. Unfortunately, very few of these variables were statistically significant in explaining differences between interventions.

We were unable to test the hypothesis that community engagement can reduce health inequalities through their impact on social inequalities due to insufficient data. In support of previous research and proposals [6,8,9], however, there was some evidence to suggest that community engagement interventions improve social inequalities (as measured by social support in seven studies: *d =* .41, 95% CI .23, .65).

We compared the effectiveness of interventions based on four different theories of change in the synthesis of effectiveness data. The results suggest that lay-delivered interventions tend to have larger effects than interventions based on empowerment or patient/consumer involvement, although this trend did not significantly explain variation in the effectiveness across studies. We propose that this association is likely to be confounded with other factors, such as intervention intensity and exposure (lay-delivered tend to be more intense, one-on-one or small group interventions, than other intervention types). For such models, we might expect to see large effects over a narrow range of outcomes, as opposed to the other theories of change models that might have smaller effects over a broader range of health and social outcomes. Unfortunately, there were insufficient data to test these relations adequately. Indeed, community engagement interventions often operate in non-linear pathways (synergies between various components and multiple outcomes) which makes evaluation complicated (compared to, for example, simple dose–response relationships). In order to assess the potentially diverse impacts of community engagement interventions, researchers need to incorporate a spectrum of outcome measures and plan long-term evaluations. Moreover, primary studies should conduct thorough process evaluations and conduct qualitative research to complement these types of evaluations as they can elucidate the ‘active ingredients’ of the intervention (and potential un-intended effects).

### Practical significance

This quantitative synthesis identified trends in the effectiveness of interventions that can be considered when designing future interventions. The following recommendations, which are based on the trends observed in the review, might be helpful for researchers and practitioners designing interventions in the future.

- Interventions that engage community members in the delivery of the intervention are particularly effective (compared with interventions that empower the community or involve members in the design of the intervention).
- Single component interventions tend to be more effective than multi-component interventions for health behaviour outcomes.
- Both universal and targeted interventions are effective, although universal interventions tend to have higher effect size estimates for health behaviour outcomes.
- Interventions that employed skill development or training strategies, or which offered contingent incentives, tended to be more effective than those employing educational strategies for health behaviour outcomes.
- Interventions involving peers, community members, or education professionals tended to be more effective than those involving health professionals for health behaviour outcomes.
- Shorter interventions tended to be more effective than longer interventions for health behaviour outcomes, although this is probably confounded by levels of exposure or intensity of contact with the intervention deliverer.
- Interventions tended to be most effective in adult populations and less effective in general populations (i.e. those that included all age groups) for health behaviour outcomes.
- Interventions tended to be most effective for health behaviour outcomes for participants classified as disadvantaged due to socioeconomic position (compared with those targeted to people on the basis of their ethnicity, place of residence, or being at/high risk). Interventions targeting participants on the basis of place of residence do not appear to be effective for health behaviour outcomes.

### Issues arising from the breadth of this review topic

This was a challenging review to undertake due to the breadth of research and perspectives it contains. As well as crossing multiple topic domains, there are also differing perspectives regarding the nature of community engagement and what should count as a community engagement intervention. Political issues loomed large, with some papers arguing for particular solutions from utilitarian and ethical positions. We navigated this uneven landscape by structuring our analysis according to the theories of change which underpin the interventions, thus transcending differences in both health topic and politics, and focusing on the intervention mechanisms which, in some situations, bring about a change in outcomes. While clinical and situational heterogeneity was inevitable and unavoidable, our conceptual framework afforded us homogeneity at the theoretical level, and any claims to generalizability must also be considered at this level (rather than, for example, probabilistic predictions).

Such broad reviews take considerable time and resource, and while there is an inevitable delay between when the searches were carried out (2011) and eventual publication, we do not think this necessarily undermines the currency of the findings presented. The theories of change around which our analyses are structured are based on enduring concepts around community engagement, some of which date from half a century and more ago. We have no reason to believe that community engagement as a theory and as a practice has undergone a fundamental shift since these theories were developed. Moreover, even if a radically new approach has been tested in a small number of studies, any effects would need to be implausibly large – as would the studies themselves – to be able to change the results of our meta-analysis (given that it is based on more than 100 studies). We are therefore confident that the results of this analysis will remain valid for many years to come.

### Issues in interpreting statistical findings

Significant statistical heterogeneity was expected in this review, and indeed the exploration of this heterogeneity was part of its design. When operating across such a wide range of topics, populations and intervention approaches, however, there is a disjunction between the conceptual heterogeneity implied by asking broad questions and the methods for analysing statistical variance that are in our ‘toolbox’ for answering them.

First, analysing the variance ‘explained’ by specific sub-groups of studies according to our conceptual framework rarely reached accepted standards for statistical significance. This is inevitable however, because conceptual homogeneity was never achieved through such a sub-division: each type of approach to engagement was observed across populations, topics, outcomes and a wide range of other unknown variables; we would therefore never reach the position of being able to say that the studies within a given sub-group differed only due to sampling error/variance. (Or that any of our sub-divisions was the only way of partitioning the studies present.) In other words, potential confounding variables or interactions amongst variables made it difficult to disentangle unique sources of variance across the studies. Second, the use of statistical significance testing in meta-analysis has itself been questioned as lacking a sound statistical basis [25,26]. While defending the practice, Mark Lipsey states that the magnitude of effect size estimates should be given greater weight in meta-analysis than the results of tests for statistical significance (and observes that if such statistical testing is wrong for meta-analysis, then it is almost certainly incorrect for most social scientific research) [27].

In the context of our analysis these debates have a clear relevance, because statistical tests for significance are unlikely to yield statistically significant findings, due to complex heterogeneity in the dataset. We are therefore left with an interpretive challenge: do we adhere strictly to the *p* > 0.05 convention before accepting that a given sub-group analysis is meaningful; or do we place more importance on the magnitude of the differences of effect size estimates between sub-groups? In this review we have attempted to plot a path somewhere between the two extremes. We have tested and reported statistical significance, but have also drawn tentative conclusions from the directions and magnitudes of effects whether or not standard statistical significance had been achieved.

A further issue for the statistical synthesis in this review relates to the comparators used in the evaluations. In the vast majority of interventions synthesised in the meta-analysis (118 out of 131; 90%), interventions were compared to a comparison condition that differed from the intervention in more ways than just community engagement. The lack of a ‘pure’ comparator in most community engagement interventions in this review could cloud our interpretation of the findings. Although we conducted a sensitivity analysis of this issue and found no difference between studies with ‘pure’ comparators versus contaminated comparators, we are unable to conclude definitively that community engagement is the ingredient necessary for intervention success. More evaluations in which community engagement is the only difference between comparison conditions are required to determine the added value of community engagement.

## Conclusions

There is solid evidence that community engagement interventions have a positive impact on a range of health and psychosocial outcomes, across various conditions. There is insufficient evidence to determine whether one particular model of community engagement is most likely to be more effective than any other.

## Endnotes

^a^Protocol available at http://www.phr.nihr.ac.uk/funded_projects/pdfs/PHR_PRO_09-3008-11_V01.pdf.

^b^Note that the research questions have been reorganised compared to the full report to facilitate presentation as a stand-alone research paper.

^c^We conducted a sensitivity analysis to test whether *d* effect size estimates based on binary data were statistically similar to *d* effect size estimates based on continuous data. Although pooled binary outcomes tended to be slightly smaller than pooled continuous outcomes, this difference was not statistically different (Q_B_ (1) = 3.03, *p* = .08).

^d^http://mason.gmu.edu/~dwilsonb/ma.html

^e^An un-weighted model, in which the weight for all studies was set to 1, was used because including study weights in the model would inflate the observed relationship between sample size (the independent variable) and effect size (the dependent variable). This is because the inverse variance study weights used in meta-analysis are largely a function of sample size.

## Appendix A: Search strategy for bibliographic databases

**Search Strategy: DoPHER (searched on 26/7/2011)**

Keyword search:

Health promotion OR inequalities AND (Aims stated AND search stated AND inclusion criteria stated)

Search Strategy: TRoPHI (searched on 16/8/2011)

“disadvantage” OR “disparities” OR “disparity” OR “equality” OR “equity” OR “gap” OR “gaps” OR “gradient” OR “gradients” OR “health determinants” OR “health education” OR “health inequalities” OR “health promotion” OR “healthy people programs” OR “inequalities” OR “inequality” OR “inequities” OR “inequity” OR “preventive health service” OR “preventive medicine” OR “primary prevention” OR “public health” OR “social medicine” OR “unequal” OR “variation”

AND

“change agent” OR “citizen” OR “community” OR “champion” OR “collaborator” OR “disadvantaged” OR “lay community” OR “lay people” OR “lay person” OR “member” OR “minority” OR “participant” OR “patient” OR “peer” OR “public” OR “representative” OR “resident” OR “service user” OR “stakeholder” OR “user” OR “volunteer” OR “vulnerable”

AND

“capacity building” OR “coalition” OR “collaboration” OR “committee” OR “compact” OR “control” OR “co-production” OR “councils” OR “delegated power” OR “democratic renewal” OR “development” OR “empowerment” OR “engagement” OR “forum” OR “governance” OR “health promotion” OR “initiative” OR “integrated local development programme” OR “intervention guidance” OR “involvement” OR “juries” OR “local area agreement” OR “local governance” OR “local involvement networks” OR “local strategic partnership” OR “mobilisation” OR “mobilization “ OR “neighbourhood committee” OR “neighbourhood managers” OR “neighbourhood renewal” OR “neighbourhood wardens” OR “networks” OR “organisation” OR “panels” OR “participation” OR “participation compact” OR “participatory action” OR “partnerships” OR “pathways “ OR “priority setting” OR “public engagement” OR “public health” OR “rapid participatory assessment” OR “regeneration” OR “relations” OR “support”

Search Strategy: Cochrane Databases (searched on 17/8/2011)

- Cochrane Database of Systematic Reviews (Cochrane Reviews)
- Database of Abstracts of Reviews of Effects (Other Reviews)
- Health Technology Assessment Database (Technology Assessments)
- NHS Economic Evaluation Database (Economic Evaluations)

“disadvantage” OR “disparities” OR “disparity” OR “equality” OR “equity” OR “gap” OR “gaps” OR “gradient” OR “gradients” OR “health determinants” OR “health education” OR “health inequalities” OR “health promotion” OR “healthy people programs” OR “inequalities” OR “inequality” OR “inequities” OR “inequity” OR “preventive health service” OR “preventive medicine” OR “primary prevention” OR “public health” OR “social medicine” OR “unequal” OR “variation”

AND

“change agent” OR “citizen” OR “community” OR “champion” OR “collaborator” OR “disadvantaged” OR “lay community” OR “lay people” OR “lay person” OR “member” OR “minority” OR “participant” OR “patient” OR “peer” OR “public” OR “representative” OR “resident” OR “service user” OR “stakeholder” OR “user” OR “volunteer” OR “vulnerable”

AND

“capacity building” OR “coalition” OR “collaboration” OR “committee” OR “compact” OR “control” OR “co-production” OR “councils” OR “delegated power” OR “democratic renewal” OR “development” OR “empowerment” OR “engagement” OR “forum” OR “governance” OR “health promotion” OR “initiative” OR “integrated local development programme” OR “intervention guidance” OR “involvement” OR “juries” OR “local area agreement” OR “local governance” OR “local involvement networks” OR “local strategic partnership” OR “mobilisation” OR “mobilization “ OR “neighbourhood committee” OR “neighbourhood managers” OR “neighbourhood renewal” OR “neighbourhood wardens” OR “networks” OR “organisation” OR “panels” OR “participation” OR “participation compact” OR “participatory action” OR “partnerships” OR “pathways “ OR “priority setting” OR “public engagement” OR “public health” OR “rapid participatory assessment” OR “regeneration” OR “relations” OR “support”

Search Strategy: Campbell Library (searched on 17/8/2011)

“disadvantage” OR “disparities” OR “disparity” OR “equality” OR “equity” OR “gap” OR “gaps” OR “gradient” OR “gradients” OR “health determinants” OR “health education” OR “health inequalities” OR “health promotion” OR “healthy people programs” OR “inequalities” OR “inequality” OR “inequities” OR “inequity” OR “preventive health service” OR “preventive medicine” OR “primary prevention” OR “public health” OR “social medicine” OR “unequal” OR “variation”

AND

“change agent” OR “citizen” OR “community” OR “champion” OR “collaborator” OR “disadvantaged” OR “lay community” OR “lay people” OR “lay person” OR “member” OR “minority” OR “participant” OR “patient” OR “peer” OR “public” OR “representative” OR “resident” OR “service user” OR “stakeholder” OR “user” OR “volunteer” OR “vulnerable”

AND

“capacity building” OR “coalition” OR “collaboration” OR “committee” OR “compact” OR “control” OR “co-production” OR “councils” OR “delegated power” OR “democratic renewal” OR “development” OR “empowerment” OR “engagement” OR “forum” OR “governance” OR “health promotion” OR “initiative” OR “integrated local development programme” OR “intervention guidance” OR “involvement” OR “juries” OR “local area agreement” OR “local governance” OR “local involvement networks” OR “local strategic partnership” OR “mobilisation” OR “mobilization “ OR “neighbourhood committee” OR “neighbourhood managers” OR “neighbourhood renewal” OR “neighbourhood wardens” OR “networks” OR “organisation” OR “panels” OR “participation” OR “participation compact” OR “participatory action” OR “partnerships” OR “pathways “ OR “priority setting” OR “public engagement” OR “public health” OR “rapid participatory assessment” OR “regeneration” OR “relations” OR “support”

## Appendix B: Data extraction and risk of bias tool

(Based on the Cochrane Risk of Bias Tool [17]).

- 1. What was the duration of the intervention?
  *In weeks; assume 4.5 weeks per month when converting*
  - Enter value in weeks (add details)
  - Duration unclear/not reported
- 2. What was the length of time between evaluation measures in weeks?
  - Time between pre-test and post-test
    *In weeks. If less than one week (e.g., a one-off session, or on two days), then use weeks = 1. Pre-test is defined as the baseline or time between start of intervention and post-test (first measurement after intervention ceases/prior to first follow-up).*
  - Time between post-test and FIRST follow-up (use if >1 follow-up)
    *In weeks*
  - Time between post-test and ONLY/FINAL follow-up
    *In weeks*
  - Measurement time unclear / not reported
- 3. Type of control group (select 1 only)
  *If more than two groups, only mark the comparison group used in effect size calculation*
  - Waitlist/delayed treatment
  - Inactive control
    *E.g., "Participants in the control group did not receive any intervention during the treatment or follow up phases"*
  - Matched data from target population, without assignment
    *The control group does not know it is a control group. Not applicable to randomised studies. E.g., comparison with area or population level statistics; comparison with prior programme participants; historical records*
  - Usual treatment/care, with assignment
  - Alternative/ placebo intervention
    *Use if the comparison group receives a different intervention to the treatment group that is not the same as usual care and which has different aims or deliverer to the main intervention*
  - Other (add details)
  - Comparator unclear / not reported
- 4. How were participants/clusters allocated to intervention and control/comparison groups?
  *Participants were allocated using an acceptable method of randomisation. NB: If method of randomisation is not stated, tick ‘yes’ but indicate this in your comments. If you have suspicions about whether methods of allocation were randomised by an acceptable method, please also indicate these here.*
  - Random
    *E.g., table of random numbers, computer-generated random sequences*
  - Partial randomisation
  - Non-random
    *E.g., date of birth, order in which participants were recruited to the study, self-selection, needs-based, matched controls*
  - Allocation unclear/not reported
- 5. SELECTION BIAS: Were participants in the two groups equivalent or adjusted in the analyses to be equivalent?
  *NB (1): Major prognostic factors are balanced between groups if the groups are drawn from similar populations and have similar socio-demographic variables and baseline values of all outcome measures. Record the extent to which your decision is supported by presented data on outcomes and/or by other information in the report (e.g. statements in text). i) Study can ‘pass’ if participants were allocated using an acceptable method of randomisation OR: ii) studies can ‘pass’ if (1) baseline values of major prognostic factors are reported for each group for virtually all participants as allocated AND if baseline values of major prognostic factors are balanced between groups in the trial OR imbalances were adjusted for in analysis.*
  - Yes – participants were allocated using acceptable method of randomisation AND groups equivalent or unimportant differences
  - Yes – baseline characteristics reported for virtually all of each group as allocated AND groups were equivalent
  - Yes – baseline characteristics reported for virtually all of each group as allocated AND imbalances between groups adjusted for in analysis
  - No - SB not avoided
  - SB unclear/not reported
- 6. Was ATTRITION BIAS avoided? (Add details)
  *Study can pass this component if: (1) the attrition rate is reported separately according to allocation group, AND if (2) the attrition rate differs across groups by less than 10% and is less than 30% overall OR baseline values of major prognostic factors were balanced between groups for all those remaining in the study for analysis. NB: For studies which are not trials, this question should simply read ‘Is the attrition rate less than 30% of the original participants?’*
  - Yes - difference in attrition rates of the groups is <10% and <30% overall
    *Attrition rate is reported separately according to allocation group AND attrition rate differs across groups by less than 10% AND is less than 30% overall*
  - Yes - ALL baseline values of prognostic factors were balanced between groups
    *Attrition rate is reported separately according to allocation group AND baseline values of prognostic factors were balanced between groups for all those remaining in the study for analysis*
  - Yes - unimportant differences between participants and drop-outs in baseline values between groups (specify)
    *Attrition rate is reported separately according to allocation group AND baseline values of major prognostic factors were balanced between groups for all those remaining in the study for analysis*
  - Yes – ITT approach or imbalances in attrition between groups adjusted for in analysis
  - No - AB not avoided
  - AB unclear/not reported
- 7. Was SELECTIVE REPORTING BIAS avoided?
  *Studies can pass this component if authors report on all outcomes they intended to measure as described in the aims of the study.*
  - Yes - SRB avoided
    *Authors report on all outcomes they intended to measure as described in the aims of the study*
  - No - SRB not avoided
- 8. Was the study sound?
  *To be sound, a study has to avoid all three of the specified types of bias Q 5–7 (selection bias, attrition bias and selective reporting bias)*
  - Sound
    *Study avoids all three of the specified types of bias (selection bias, follow-up bias and selective reporting bias)*
  - Not sound
- 9. Multiple treatment or comparison arms?
  *Does the study have more than two groups?*
  - Yes - multiple treatment or comparison arms
  - No - only one intervention and one comparison group
- 10. Outcomes
  - Health outcomes
    *Only extract health status outcomes if a health behaviour has also been measured*
    - Alcohol abuse
    - Antenatal (prenatal) care
    - Breastfeeding
    - Cancer screening
    - Cardiovascular disease
    - Child abuse prevention
    - Child illness and ill health
      *This also includes birth outcomes (e.g. low birthweight LBW)*
    - Drug abuse
    - Healthy eating
    - Hypertension
    - Immunisation
    - Injury/safety
    - Mental health
    - Neighbourhood renewal/regeneration
    - Obesity/weight status
    - Parenting
      *Includes child development training/education*
    - Physical activity
    - Sexual health related to teenage pregnancy
      *Outcomes incl. pregnancy, contraceptive use/safe sex practices, abstinence etc.*
    - Smoking cessation
    - Smoking/tobacco prevention
    - Other not captured above
  - Community outcomes
    - Community outcome
  - Engagee personal outcomes
    - Engagee Empowerment
    - Engagee Self-esteem
    - Engagee Skills
    - Engagee Social support/capital/inclusion
    - Engagee Health
  - PH participant personal outcomes
    - PH participant Self-efficacy
    - PH participant Social support/capital
- Outcome classification codes
  - Immediate post-test (Required)
    *Mark if the data were measured at immediate post-test (i.e., the first measure taken after the intervention is completed). Effect sizes must be coded as either "Immediate post-test" or "Final follow-up".*
  - First follow-up (if more than one) (1FU)
  - ONLY/ Final follow-up (O/FFU)
    *Mark if the data were measured at follow-up (also called delayed post-test). This should be the final measure taken after the intervention is completed, regardless of amount of time lapsed or number of other measurements taken between completion and final measurement. Effect sizes must be coded as either "Immediate post-test" or "Final follow-up".*
  - Effect 1: Favours intervention (Required)
    *Effect sizes must be coded as either "favours intervention" or "favours control"*
  - Effect 2: favours control (Required)
    *Effect sizes must be coded as either "favours intervention" or "favours control"*
  - Health behaviour: Actions
    *Mark if the outcome is an observable behaviour (i.e. things people do), such as drinking, smoking, cooking, physical activity Or a measure of intake such as amount of fruit consumed or cigarettes smoked*
  - Status 1: Physiological consequences
    *Only extract if health behaviours are also extracted - unless measure of teenage pregnancy. These are not something that you do, they are the consequences of your behaviours. Consequences of behaviours (metabolic and physiological risk factors and related biomarkers), such as pregnancy, blood pressure, cotinine levels, cholesterol, BMI*
  - Status 2: Final health outcomes
    *Only extract if health behaviours are also extracted. Final health outcomes: diagnosis, morbidity and mortality associated with relevant diseases. Incl. clinical diagnoses such as obesity, CVD, diabetes, cancer*
  - Calculation required imputation
    *Mark if not all of the necessary data were explicitly reported and some imputation was required (e.g., assuming equal numbers in treatment & control groups if exact n not stated; imputing values from "p < ")*
  - Measure is self-report
    *Mark if the effect size is calculated from data that was measured using self-report.*
  - Sub-group analysis
- Sub-sample health inequality
  *This refers to the PROGRESS+ group of the sub-sample for which the effect size is calculated.*
  - Ethnicity
  - Socio-economic status/position
    *Income, means tested benefits/welfare, affluence measures, deprived area, classification as ‘low’ SEP*
  - Occupation/employment status
  - Education
    *Years in and/or level of education attained, school type. Includes high school dropouts*
  - Place of residence
  - Sexual orientation
  - Social capital
  - Gender
  - Religion
  - Age
  - Marital status/ family composition
  - Disability
  - 'At-risk' or 'high risk' youths, incl. homeless/runaways
  - Substance abuse (e.g., injecting drug users)
    *Includes intravenous/injecting drug users and other chronic or hard drug abusers. Does not include minor recreational or experimental drug use.*
  - Teenage parents/ pregnant teens
  - Multiple health inequalities

Other vulnerable groups

## Additional file

Additional file 1:
**Results of Risk of Bias assessment.**
