# Supplementary material for: The effectiveness of community engagement in public health interventions for disadvantaged groups: a meta-analysis
Source: BMC Public Health. 2015 Feb 12;15:129. doi: 10.1186/s12889-015-1352-y (PMC4374501; doi:10.1186/s12889-015-1352-y)
Supplement: Additional file 1: — Results of Risk of Bias assessment. [file 12889_2015_1352_MOESM1_ESM.docx]

Results of Risk of Bias assessment

| Study | Was selection bias avoided? | Was attrition bias avoided? | Was selective reporting bias avoided? | Was the study sound overall? |
| --- | --- | --- | --- | --- |
| Anand (2007) | + | + | + | + |
| Anderson (2005) | + | + | - | - |
| Andrews (2007) | + | + | + | + |
| Arlotti (1998) | + | + | + | + |
| Aseltine (2000) | ? | + | + | - |
| Auld (1998) | + | + | + | + |
| Auslander (2002) | + | + | + | + |
| Avila (1994) | + | - | + | - |
| Ayala (2010) | + | + | - | - |
| Balcazar (2010) | + | + | + | + |
| Banks (2009) | + | - | - | - |
| Baranowski (1990) | - | - | + | - |
| Barnes (1999) | + | + | + | + |
| Barnes-Boyd (2001) | - | - | - | - |
| Becker (2005) | + | + | + | + |
| Beech (2003) | + | + | + | + |
| Black (2010) | + | + | + | + |
| Botvin (1995) | - | + | + | - |
| Britto (2001) | + | - | + | - |
| Brownson (1996) | + | - | + | - |
| Brownson (2004) | + | - | + | - |
| Brownson (2005) | + | - | + | - |
| Buller (1999) | + | + | + | + |
| Campbell (1999) | + | + | + | + |
| Campbell (2002) | + | ? | + | - |
| Campbell (2004) | + | + | + | + |
| Caulfield (1998) | + | - | - | - |
| Chapman (2004) | + | + | + | + |
| Cherry (1998) | + | + | + | + |
| Cochrane (2008) | + | + | + | + |
| Conway (2004) | + | + | + | + |
| Cox (1995) | ? | ? | + | - |
| Daniel (1999) | - | ? | - | - |
| Davidson (1994) | - | ? | + | - |
| Davis (1993) | ? | ? | - | - |
| Davis (1995) | + | + | + | + |
| Dedobbeleer (2001) | + | + | + | + |
| Duncan (2000) | + | + | + | + |
| Elder (1993) | + | + | + | + |
| Elder (2005) | + | + | - | - |
| Ernst (1999) | + | + | + | + |
| Faridi (2010) | + | - | + | - |
| Fisher (1998) | + | ? | - | - |
| Fitzgibbon (2005) | + | + | + | + |
| Foerster (1998) | - | - | + | - |
| Fried (2004) | - | - | + | - |
| Gadomski (2006) | + | + | + | + |
| Gottfredson (1996) | + | + | + | + |
| Government (2006) | - | - | - | - |
| Graham (1992) | + | + | + | + |
| Grummer-Strawn (1997) | ? | ? | + | - |
| Hancock (2001) | + | ? | - | - |
| Hanlon (2002) | + | ? | + | - |
| Harmon (1995) | + | + | + | + |
| Havas (1998) | - | + | + | - |
| Hayashi (2010) | + | + | + | + |
| Hecht (2003) | + | + | + | + |
| Hunter (2004) | + | + | + | + |
| Johnson (1993) | + | + | + | + |
| Julnes (1994) | - | + | + | - |
| Karanja (2010) | + | + | + | + |
| Kim (2008) | + | + | + | + |
| Kistin (1994) | + | + | + | + |
| Kloek (2006) | + | + | + | + |
| Krieger (1999) | + | + | + | + |
| Kumpusalo (1996) | ? | - | + | - |
| Lewis (1993) | + | - | + | - |
| Lindenberg (2002) | + | + | + | + |
| Lindqvist (1999) | + | + | + | + |
| Long (1995) | + | + | + | + |
| LoSciuto (1996) | + | + | + | + |
| LoSciuto (1999) | + | + | + | + |
| Lupton (2002) | - | + | + | - |
| Luster (1996) | + | - | + | - |
| Macaulay (1997) | - | - | + | - |
| Malchodi (2003) | + | - | + | - |
| Marcenko (1996) | + | + | + | + |
| McAlister (1992) | + | - | + | - |
| McInnes (1998) | - | + | + | - |
| McNabb (1993) | + | + | + | + |
| McNabb (1997) | + | - | + | - |
| Mendoza (2009) | - | - | + | - |
| Miller-Heyl (1998) | + | + | + | + |
| Nafziger (2001) | + | + | + | + |
| Norr (2003) | + | + | + | + |
| O'Loughlin (1999) | + | - | + | - |
| Parsons (1992) | - | - | - | - |
| Perry (1996) | + | + | + | + |
| Platt (2003) | - | ? | - | - |
| Poland (1992) | + | - | + | - |
| Poston (2001) | + | - | + | - |
| Pugh (2001) | + | + | + | + |
| Pugh (2002) | + | + | + | + |
| Reijneveld (2003) | + | + | + | + |
| Resnicow (1992) | + | - | + | - |
| Resnicow (2004) | + | + | + | + |
| Rhodes (2005) | + | + | - | - |
| Robinson (2003) | + | + | + | + |
| Rodewald (1999) | + | + | + | + |
| Rose (1992) | + | + | + | + |
| Schafer (1998) | - | - | + | - |
| Schensul (2009) | + | - | + | - |
| Schinke (2000) | + | + | + | + |
| Schorling (1997) | + | + | + | + |
| Schuler (2002) | + | + | + | + |
| Schwarz (1993) | - | + | + | - |
| Secker-Walker (2000) | + | - | + | - |
| Shaw (1997) | ? | + | + | - |
| Shaw (1999) | + | - | + | - |
| Shelley (2008) | + | + | + | + |
| Silver (1997) | + | + | + | + |
| Simmons (1998) | + | + | + | + |
| Solomon (2000a) | + | + | + | + |
| Solomon (2000b) | + | + | + | + |
| Sorensen (2005) | + | + | + | + |
| St James (1999) | + | ? | - | - |
| Staten (2004) | + | - | + | - |
| Valente (2006) | ? | + | + | + |
| Vogler (2002) | + | ? | + | - |
| Voorhees (1996) | + | + | + | + |
| Watkins (1994) | ? | - | + | - |
| Weiss (1998) | + | ? | + | - |
| Wiist (1990) | + | + | + | + |
| Wilcox (2007) | ? | - | + | - |
| Winkleby (2004) | + | + | + | + |
| Witmer (2004) | ? | ? | + | - |
| Woodruff (2002) | + | + | + | + |
| Wright (1997) | + | ? | - | - |
| Yanek (2001) | + | + | + | + |
| Zavela (2004) | + | ? | + | - |
| Zhou (2003) | ? | + | - | - |
